# SEOM clinical guidelines on venous thromboembolism (VTE) and cancer (2023)

**DOI:** 10.1007/s12094-024-03605-2

**Published:** 2024-08-07

**Authors:** Laura Ortega Morán, Francisco José Pelegrín Mateo, Rut Porta Balanyà, Jacobo Rogado Revuelta, Silverio Ros Martínez, José Pablo Berros Fombella, Elena María Brozos Vázquez, Natalia Luque Caro, José Muñoz Langa, Mercedes Salgado Fernández

**Affiliations:** 1 Hospital General Universitario Gregorio Marañón, Instituto de Investigación Sanitaria Gregorio Marañón, Madrid, Spain; 2 Hospital de la Santa Creu i Sant Pau, Barcelona, Spain; 3 Hospital Universitari Dr. Josep Trueta, ICO Girona, Girona, Spain; 4 Hospital Universitario Infanta Leonor, Madrid, Spain; 5 Hospital Universitario Virgen de la Arrixaca, Murcia, Spain; 6 Hospital Universitario San Agustín, Avilés, Asturias Spain; 7 Complexo Hospitalario Universitario A Coruña, A Coruña, Spain; 8 Hospital Universitario Jaén, Jaén, Spain; 9 Hospital Arnau de Vilanova, Valencia, Spain; 10 Complexo Hospitalario Universitario de Ourense, Ourense, Spain

**Keywords:** Venous thromboembolism, Cancer, Anticoagulation, Guidelines, Low-molecular-weight heparins, Direct oral anticoagulants

## Abstract

The Spanish Society of Medical Oncology (SEOM) last published clinical guidelines on venous thromboembolism (VTE) and cancer in 2019, with a partial update in 2020. In this new update to the guidelines, SEOM seeks to incorporate recent evidence, based on a critical review of the literature, to provide practical current recommendations for the prophylactic and therapeutic management of VTE in patients with cancer. Special clinical situations whose management and/or choice of currently recommended therapeutic options (low-molecular-weight heparins [LMWHs] or direct-acting oral anticoagulants [DOACs]) is controversial are included.

## Introduction

Patients with cancer face a significantly higher risk of venous thromboembolism (VTE), with ninefold higher than in de general population making it the second leading cause of preventable death amongst cancer patients [1]. Furthermore, those who develop VTE at diagnosis of cancer or within a year of diagnosis have a worse disease prognosis compared with cancer patients without VTE [2]. On the other hand, cancer patients who are undergoing cancer treatment have both an increased risk of thrombotic recurrences and a twofold risk of suffering serious bleeding [2]. In addition, people with cancer who undergo surgical resection are at significantly higher risk of peri- and post-operative VTE than patients who undergo surgery for non-malignant diseases [3].

Several factors contribute to this heightened risk, including patient-specific characteristics, tumour-related factors and treatment modalities. A wide variety of risk factors related to the patient have been identified, such as advanced age (over 65 years old), hospitalisation and prolonged immobilisation, previous history of VTE, familial or acquired coagulation disorders (thrombophilias), etc. Furthermore, tumour cells express and release procoagulant molecules (e.g. tissue factor, cancer procoagulant factor, the mucin of carcinoma, interleukin-1 [IL-1], tumour necrosis factor-alpha [TNF-α] and vascular endothelial growth factor [VEGF]) that, in addition to activating the coagulation cascade, interact with platelets and cells of the immune system, especially the monocyte-macrophage system, producing a state of hypercoagulability in cancer patients [4]. Moreover, cancer surgery, anticancer drugs and central venous catheters (CVC) can activate the endothelium cells, leading to endothelial dysfunction and facilitating the formation of thrombus or haemorrhage [5].

Although there are several scoring systems that attempt to assess the risk of a first VTE event, their usefulness is limited in primary thromboprophylaxis strategies, due to the low penetration of their use by healthcare professionals.

In a significant percentage of cancer patients, VTE occurs in special situations that make the management and treatment of thrombosis more complicated. This is usually associated with greater uncertainty and lack of evidence about the optimal approach in such circumstances. These special situations comprise patients with temporary thrombocytopenia due to chemotherapy and/or radiation therapy or sustained chronic thrombocytopenia, high risk of bleeding related to the cancer or its treatment or with the coexistence of active bleeding; patients who receive new non-cytostatic treatments (targeted therapies, antiangiogenics, immunotherapy) and patients with severe kidney failure. However, only a few randomised clinical trials (RCTs) on cancer-associated thrombosis (CAT) have been published in the last 2 decades.

New data have emerged in recent years, shedding light on this complex relationship. As a result, the number of studies specifically focussing on this population has significantly increased. Whilst low-molecular-weight heparins (LMWH) have traditionally been the standard treatment for CAT, the exploration of novel anticoagulants has opened up new avenues for therapeutic options.

This updated edition of the SEOM guidelines on thrombosis and cancer provides clinicians with a comprehensive review of these recent findings, along with their respective levels of evidence, and can therefore be a valuable resource in clinical practise.

## Methodology

These guidelines are an update of the previous clinical guidelines on VTE and cancer published in 2020 [6] based on a systematic review of relevant published studies and with the consensus of ten treatment expert oncologists from the Thrombosis Section of the SEOM. The Expert Panel met via webinar and corresponded through e-mail. Based on the consideration of the evidence, the authors were asked to contribute to the development of the guidelines, provide critical review and conclude with recommendations.

Members of the Expert Panel were responsible for reviewing and approving the penultimate version of the guidelines, which was then sent to an external review panel of two experts designated by SEOM for final review.

The Infectious Diseases Society of America–US Public Health Service Grading System for ranking recommendations in clinical guidelines [7] has been used to assign levels of evidence and grades of recommendation (Table 1).

**Table 1:** Levels of evidence and grades of recommendation from the Infectious Diseases Society of America [7]

Category, grade	Definition
Levels of evidence	
I	Evidence from at least one large randomised, controlled trial of sound methodological quality (low potential for bias) or meta-analyses of well-conducted randomised trials without heterogeneity
II	Small randomised trials or large randomised trials with a suspicion of bias (lower methodological quality) or meta-analyses of such trials or of trials with proven heterogeneity
III	Prospective cohort studies
IV	Retrospective cohort studies or case–control studies
V	Studies without control group, case reports, experts’ opinions
Strength of recommendation	
A	Strong evidence for efficacy with a substantial clinical benefit; strongly recommended
B	Strong or moderate evidence for efficacy, but with limited clinical benefit; generally recommended
C	Insufficient evidence of efficacy or benefit does not outweigh the risk or the disadvantages (adverse events, costs, etc.); optional
D	Moderate evidence against efficacy or for adverse outcome; generally not recommended
E	Strong evidence against efficacy or for adverse outcome; never recommended

This update aims to incorporate the recently published scientific evidence in this field and provide practical recommendations for the management of CAT.

### Prophylaxis OF VTE

1. Prophylaxis of VTE in patients with cancer hospitalised for an acute medical illness

Hospitalisation is associated with an increased risk of VTE in patients with cancer [8]. In addition to experiencing reduced mobility, many hospitalised patients with cancer have additional risk factors for VTE, such as infection or other acute medical conditions or advanced age [9].

Three large randomised phase III trials on prophylactic anticoagulation in hospitalised patients for an acute medical illness demonstrated a risk reduction for VTE following treatment with LMWH or fondaparinux compared with placebo, without a significant increase in bleeding events [10–12] (Table 2). In these studies, only 5% to 15% of patients had cancer. In this subgroup of patients, thromboprophylaxis demonstrated a reduction in VTE events only in the MEDENOX and PREVENT clinical trials. In the ARTEMIS study, a higher incidence rate of VTE events was observed in the fondaparinux group (17%) than in the placebo group (3.9%). This contradictory result has not been well understood: different VTE-risk cancer populations, type of heparin (fondaparinux), etc.

**Table 2:** Randomised clinical trials assessing pharmacological prophylaxis of venous thromboembolism in hospitalised acutely medically ill patients

RCT	n	Cancer patients (n, %)	Study drug/schedule	All patients		Cancer subgroup	
				VTE events	MB	VTE events	CRB or MB
ARTEMIS [Cohen AT, BMJ. 2006]	849	HC: 131 (15.4%)	Fondaparinux sc (2.5 mg/24 h) for 6–14 days vs Placebo No extended prophylaxis	5.6 vs 10.5% RR: 0.47 *p* = 0.029	0.2 vs 0.2% *p* = NS	17.0 vs 3.9% RR: 4.3	NR
MEDENOX [Samama MM, N Engl J Med. 1999]	738	HC: 101 (13.7%)	Enoxaparin sc (40 mg/24 h) for 6–14 days vs Placebo No extended prophylaxis	5.5 vs 14.9% RR: 0.37 *p* < 0.001	1.7 vs 1.1% *p* = NS	9.7 vs 19.5%, RR: 0.50	NR
PREVENT [Leizorovicz A, Circulation. 2004]	3706	HC: 190 (5.1%)	Dalteparin sc (5000 UI/24 h) for 14 days vs Placebo No extended prophylaxis	2.8 vs 5.0% RR: 0.55 *p* = 0.0015	0.5 vs 0.2% *p* = NS	3.1 vs 8.3% RR: 0.37	NR
EXCLAIM [Hull RD, Ann Intern Med. 2010]	5963	HC: 818 (13.7%) AC: 100 (1.7%) (*)	Enoxaparin sc (40 mg/24 h) for 10 ± 4 days, then enoxaparin sc (40 mg/24 h) for additional 28 ± 4 days vs Enoxaparin sc (40 mg/24 h) for 10 ± 4 days *Extended prophylaxis for enoxaparin*	2.5 vs 4.0% ARD: − 1.53% (95% CI − 2.54% to − 0.52%)	0.8 vs 0.3% ARD: 0.51% (95% CI 0.12% to 0.89%)	OR: 0.71 (95% CI 0.33–1.53)	NR
ADOPT [Goldhaber SZ, N Engl J Med. 2011]	6528	HC: 632 (9.7%) AC: 211 (3.2%)	Oral apixaban (2.5 mg/12 h) for 30 days vs Enoxaparin sc (40 mg/24 h) for 6–14 days *Extended prophylaxis for apixaban*	2.71 vs 3.06% RR: 0.87 *p* = 0.44	0.47 vs 0.19% RR: 2.58 *p* = 0.04	NR	NR
MAGELLAN [Cohen AT, N Engl J Med. 2013]	8101	HC: 1378 (17.0%) AC: 592 (7.3%) (*)	Oral rivaroxaban (10 mg/24 h) for 35 ± 4 days vs Enoxaparin sc (40 mg/24 h) for 10 ± 4 days *Extended prophylaxis for rivaroxaban*	4.4 vs 5.7% RR: 0.77 *p* = 0.02	4.1 vs 1.7% RR: 2.5 *p* < 0.001	6.9 vs 7.9% OR: 0.87 (95% CI 0.51–1.48)	5.7 vs 1.8% OR: 3.2 (95% CI 1.6–6.5)
APEX [Cohen AT, N Engl J Med. 2016]	7513	HC: 909 (12.1%) AC: 37 (0.49%) (*)	Oral betrixaban (80 mg/24 h) for 35–42 days vs Enoxaparin sc (40 mg/24 h) for 10 ± 4 days	5.3 vs 7.0% RR: 0.76 *p* = 0.006	0.7 vs 0.6% *p* = 0.55	5.7 vs 6.2% OR: 0.92 (95% CI 0.54–1.58)	2.8 vs 1.9% OR: 1.44 (95% CI 0.62–3.36)
MARINER [Spyropoulos AC; N Engl J Med. 2018]	12,019	HC: 1022 (8.5%) AC: excluded (**)	Oral rivaroxaban (10 mg/24 h) for 45 days vs Placebo for 45 days after discharge *Extended prophylaxis for rivaroxaban*	0.83 vs 1.1% RR: 0.76 *p* = 0.14	0.28% vs 0.15% RR: 1.88 *p* = NS	1.02 vs 1.31 OR: 0.78 (95% CI 0.25–2.47)	3.7 vs 2.1% OR: 1.8 (95% CI 0.9–3.9)

*AC* active cancer, *ARD* absolute risk difference, *CI* confidence interval, *CRB* clinically relevant bleeding, *HC* history of cancer (active and remote cancer), *MB* major bleeding, *n* sample size, *NR* not reported, *NS* not significant, *OR* odds ratio, *p* significance level, *RCT* randomised clinical trials, *RR* relative risk, *sc* subcutaneous, *vs* versus, *VTE* venous thromboembolism

(*) Excludes intracranial neoplasm or metastasis. (**) Excludes all active cancers

Carrier et al. published a meta-analysis of 307 cancer patients out of 5134 total study subjects (6%) from these three RCTs, showing no significant reduction in the incidence of VTE in cancer patients treated with prophylaxis [relative risk (RR) 0.91; 95% confidence interval (CI) 95% 0.21–4.0; I^2^: 68%] [13]. The apparent lack of efficacy of thromboprophylaxis in hospitalised patients with cancer could be explained by a number of reasons: heterogeneity between studies was apparent (I^2^: 68%); the low number of patients included in the meta-analysis (307 patients in total), reflecting inadequate power to detect a small relative benefit; the different VTE-risk cancer populations (none of the studies included randomised patients according to their cancer status or stratified patients according to their underlying risk of VTE events); the recommended thromboprophylactic fixed doses might not be optimal for hospitalised patients with cancer; fondaparinux may be less efficacious compared with dalteparin or enoxaparin in cancer cohorts (ARTEMIS study), etc.

Despite the routine use of thromboprophylaxis during hospitalisation, VTE after hospital discharge remains an important preventable cause of death [14]. The initial study investigating extended prophylaxis in medical patients, the EXCLAIM study [15], randomly assigned patients to 10 vs 28 days of prophylactic-dose LMWH. Although EXCLAIM found extended thromboprophylaxis beneficial in certain high-risk subsets, it also noted a significant increase in major bleeding (MB).

Several RCTs have compared extended oral direct factor Xa inhibitor anticoagulants (DOACs) (apixaban, rivaroxaban and betrixaban) with standard-course LMWH prophylaxis in acutely ill medical patients, showing that extended thromboprophylaxis can reduce the incidence of VTE but this is counterbalanced by an increased risk of haemorrhage (Table 2).

Two published meta-analyses of these RCTs suggest the same conclusion [16, 17]: extended-course DOAC thromboprophylaxis in hospitalised acutely ill medical (non-surgical) patients is associated with a significant decrease in total and symptomatic VTE compared with standard-course LMWH but is associated with an increase in both total and MB, suggesting against the routine use of extended prophylaxis in the general population of hospitalised medical patients.

Osataphan et al. conducted a systematic review and meta-analysis to delineate the benefit of extended thromboprophylaxis in preventing VTE and the risk of bleeding specifically in hospitalised patients with cancer [18]. Four RCTs reported the outcomes of extended thromboprophylaxis in 3655 medically ill patients with active (20%) or history of cancer (80%). A pooled analysis of cancer patients showed no differences between the rates of VTE events in the extended prophylaxis group when compared with standard prophylaxis [odds ratio (OR) 0.85; 95% CI 0.61–1.18; I^2^: 0%). However, the risk of clinically relevant bleeding was higher in the extended-duration thromboprophylaxis group (OR 2.11; 95% CI 1.33–3.35; I^2^: 8%).

In conclusion, extended thromboprophylaxis in hospitalised medically ill patients with cancer was not associated with a reduced rate of VTE events but was associated with an increased risk of bleeding.

To date, no randomised phase III trials have evaluated inpatient thromboprophylaxis in a cancer-only population. Randomised studies should therefore be designed in such a setting with a higher number of patients and stratifications according to VTE risk and LMWH dose adjustment to obtain definitive conclusions.

#### Recommendations

These recommendations were formulated by extrapolating the best available data from hospitalised medical patients, including patients with cancer.

- Pharmacological thromboprophylaxis should be considered in hospitalised cancer patients with acute medical illness or reduced mobility in the absence of bleeding or other contraindications (level of evidence: I; grade of recommendation: B).
- The preferred agents are LMWHs due to their favourable safety profile (level of evidence: I; grade of recommendation: B).
- At present, the authors do not recommend extended thromboprophylaxis amongst hospitalised medically ill patients with cancer, highlighting the need for an individualised approach to management (level of evidence: II; grade of recommendation: E).

2. Prophylaxis of VTE in surgical cancer patients

An assessment of the risk of thrombosis and bleeding should be carried out before any surgical procedure, including cancer surgery.

VTE is a common complication in cancer patients undergoing surgery, with a twofold or greater increased risk of deep vein thrombosis (DVT) and four times more risk of a fatal post-operative pulmonary embolism (PE) compared to the non-cancer population. VTE accounts for 10% of post-operative early mortality [19, 20].

The risk of VTE depends on specific factors of the patient, the tumour and the surgical procedure, the type and duration of the anaesthesia, the advanced age of the subject, the residual disease after surgery, obesity, advanced stages of disease, prolonged immobility (more than 3 days), and the most important background: thromboembolism [19, 20].

Several RCTs and multiple meta-analysis [21] have demonstrated the benefit of perioperative pharmacological thromboprophylaxis over no prophylaxis or placebo in patients with cancer undergoing surgical intervention, unless there is a contraindication. There were no significant differences in risk of PE, DVT, mortality or bleeding between LMWH and unfractionated heparins (UFH). However, LMWHs have a lower risk of heparin-induced thrombocytopenia and a more convenient administration schedule, which makes them an attractive first-choice agent. No data supports the superiority of any LMWH over any other. The 2018 Cochrane review also compared LMWH with fondaparinux based on three RCTs in the perioperative setting. The two agents did not differ significantly regarding the risk of VTE or MB, but the certainty of evidence was low [21].

Mechanical methods (intermittent pneumatic compression or graduated compression stockings) can be added to pharmacological prophylaxis (LMWH or UFH) to increase efficacy in preventing DVT and/or PE without increasing the incidence of MB [22–24]. However, combined prophylaxis is rarely used in daily clinical practise in oncology patients.

These meta-analyses and small RCTs in cancer surgical patients have shown the superiority of pharmacological prophylaxis over mechanical methods alone in reducing the occurrence of VTE complications [25, 26]. Therefore, mechanical methods should not be used as the sole treatment strategy unless there are contraindications for pharmacological prophylaxis (active bleeding or high-risk bleeding).

In clinical practise, prophylaxis is traditionally continued for at least 7–10 days. Nevertheless, the risk of VTE persists several weeks after abdominopelvic cancer surgery. Up to 40% of VTE events may occur later than 21 days from the surgical intervention [20], and more than 50% may do so after hospital discharge [24]. The benefit of extended thromboprophylaxis in high-risk patients has been assessed in multiple RCTs and meta-analyses.

In 2002, Bergqvist et al. reported the results of a double-blind trial with 332 patients undergoing curative surgery for abdominal or pelvic cancer [25]. Patients received enoxaparin 40 mg daily for 6 to 10 days and were then randomly assigned to receive either enoxaparin or placebo for another 21 days. The primary endpoint was the incidence of VTE between days 25 and 31. The rates of VTE at the end of the study were 12.0% in the placebo group and 4.8% in the enoxaparin group (significance level [*p*] = 0.02). This difference persisted at 3 months (13.8% vs 5.5%; *p* = 0.01). There were no significant differences in the rates of bleeding or other complications during the double-blind or follow-up periods. Other clinical trials with similar designs have achieved similar results.

In 2014, Vedovati et al. assessed the benefit of extended thromboprophylaxis in laparoscopic surgery [26]. A total of 225 patients undergoing laparoscopic surgery for colorectal cancer were randomised to 1 week or 4 weeks of thromboprophylaxis with LMWH. The VTE incidence rate at 4 weeks after surgery was 9.7% in the 1-week arm compared to 0% in the extended treatment arm (*p* = 0.001), with a similar incidence of bleeding and mortality rates. Similar efficacy results were observed at 3 months (VTE incidence: 9.7% vs 0.9%; *p* = 0.005).

In 2016, Fagarasanu et al. [27] published a systematic review and meta-analysis of seven randomised and prospective studies comprising 4807 patients. Extended thromboprophylaxis decreased the incidence of all VTEs (RR 0.44; 95% CI 0.28–0.70) without a significant difference in the incidence of MB (RR 1.19; 95% CI 0.47–2.97).

Another systematic review and meta-analysis published in 2017 by Guo et al. also concluded that extended pharmacological prophylaxis with LMWH significantly reduces VTE after cancer surgery with a non-significant increase in bleeding complications [28].

The 2019 update of the Cochrane review [29] concluded that prolonged thromboprophylaxis with LMWH significantly reduces the risk of VTE compared to thromboprophylaxis during hospital admittance without increasing bleeding complications or mortality.

More recently, a meta-analysis and literature review published in 2021 by Knoll et al. concluded that extended thromboprophylaxis in patients undergoing abdominopelvic cancer surgery was associated with a significant reduction in the incidence of clinical VTE (1.0% vs 2.1%; RR 0.48, 95% CI 0.31–0.74; I^2^ = 0), without a significant increase in clinically relevant bleeding (4.0% vs 4.9%; RR 1.0, 95% CI 0.66–1.5, I_2_ = 0) [30].

Based on the data described above, there is additional evidence that extending the administration of LMWH for 30 days after the day of surgery reduces the risk of VTE for patients undergoing either laparoscopic or open surgery for abdominal and pelvic cancer. Moreover, this reduction in VTE was not associated with an increase in bleeding complications.

In the past 2 years, two RCTs have found evidence of the safety and efficacy of two DOACs for extended post-operative thromboprophylaxis [31, 32]. The double-blind PROLAPS-II trial compared rivaroxaban with placebo in 582 patients undergoing laparoscopic surgery for colorectal cancer [31]. Study treatment began 7 days (± 2 days) after surgery and continued for 3 weeks. All patients received LMWH from the time of surgery to the start of the study treatment. The primary outcome (a composite of symptomatic VTE, asymptomatic DVT or VTE-related death in the first 28 days after surgery) occurred in 1% of patients in the rivaroxaban arm and 3.9% of patients in the placebo arm (*p* = 0*.*03). MB occurred in 0.7% of patients in the rivaroxaban arm and zero patients in the placebo arm.

Extended post-operative thromboprophylaxis with apixaban vs enoxaparin was evaluated in a randomised, open-label trial of 400 patients undergoing surgery for suspected or confirmed gynaecologic cancer [32]. Patients received heparin on the first post-operative day, with random assignment to apixaban or enoxaparin within the first week after surgery, when deemed safe by the operating surgeon. Study treatment was provided for 28 days, and patients were followed for a total of 90 days. MB occurred in one patient in each study arm. Clinically relevant non-major bleeding (CRNMB) occurred in 5.4% of patients in the apixaban arm and 9.7% of patients in the enoxaparin arm (*p* = 0.11). VTE, a secondary outcome, occurred in 1% of patients in the apixaban arm and 1.5% of patients in the enoxaparin arm (*p* = 0.68).

#### Recommendations

- In the absence of active bleeding, high bleeding risk or other contraindications, all cancer patients undergoing major surgical intervention should be offered pharmacological thromboprophylaxis with either LMWH, the preferred agents, or UFH (level of evidence: I; grade of recommendation: A).
- Prophylaxis should be started before surgery or as soon as possible in the post-operative period. Patients should receive at least 7–10 days of prophylaxis (level of evidence: I; grade of recommendation: A).
- Mechanical methods may be added to pharmacological prophylaxis in high-risk patients but should not be used as monotherapy unless pharmacological prophylaxis is contraindicated (level of evidence: II; grade of recommendation: C).
- Patients undergoing major open or laparoscopic abdominal or pelvic cancer surgery with high-risk features, such as restricted mobility, obesity or history of VTE, or with additional risk factors, should be considered for extended thromboprophylaxis with LMWH for up to 4 weeks (level of evidence: I; grade of recommendation: A).
- Alternatively, those patients who are candidates for extended thromboprophylaxis after surgery may be offered prophylactic doses of rivaroxaban or apixaban after an initial period of LMWH. Additional data from more RCTs are needed to strengthen this recommendation (level of evidence: II; grade of recommendation: B).

3. Prophylaxis of VTE in ambulatory cancer patients during systemic therapy

More than 70% of VTEs occur in the outpatient setting. Systemic treatment has been identified as an independent risk factor for VTE in cancer patients. However, routine thromboprophylaxis is currently not recommended for all patients receiving systemic treatment. The most recent RCTs on ambulatory thromboprophylaxis have incorporated the use of thrombotic risk assessment models (RAM), such as the Khorana score, to select patients at higher risk of developing VTE at the initiation of systemic therapy [33–35] (Table 3).

**Table 3:** Randomized studies of thromboprophylaxis in cancer patients with outpatient treatment

Study	*n*	Type of tumour	Treatment and dosage	Primary endpoint	CAT	MB	Minor bleeding or other non-major bleeding
PROTECHT [33]	1150	Lung, digestive, breast, ovarian Moderate-high CAT risk	Nadroparin 3800U/24 h 4 months vs no thromboprophylaxis	Thrombosis-related	2.0% vs 3.9% (VTE + ATE) *p* = 0.02	0.7% vs 0% *p* = 0.18	7.4% vs 7.9% *p* = NS
FRAGEM [34]	123	Pancreas High CAT risk	Dalteparin 200U/kg/24 h 4 weeks followed 150U/kg//24 h 8 weeks vs no thromboprophylaxis	Thrombosis-related	3.4% vs 23.0% *p* = 0.002	3.4% vs 3.2% *p* = NS	9% vs 3%
CONKO-004 [35]	312	Pancreas High CAT risk	Enoxaparin 1 mg/kg/24 h, 3 months, followed 40 mg/24 h 3 months vs no thromboprophylaxis	Thrombosis-related	1.2% vs 9.9% *p* = 0.001	4.8% vs 3.3% *p* = 1.0	NR
SAVE ONCO [36]	3212	Lung, digestive, kidney, ovary Moderate–high CAT risk	Semuloparin 20 mg/24 h until a change of CT regimen vs no thromboprophylaxis	Thrombosis-related	1.2% vs 3.4% *p* < 0.001	1.2% vs 1.2% *p* = NS	Clinically relevant non-major bleeding 1.6% vs 0.9%, OR 1.86; *p* = NS Clinically relevant bleeding 2.8% vs 2.0%, OR 1.41; *p* = NS
PRODIGE [37]	186	Glioma High CAT risk	Dalteparin 5000 IU/24 h 6 months vs no thromboprophylaxis	Thrombosis-related	3.0% vs 0.0% *p* = NS	3.0% vs 0.0% *p* = NS	NR
FRAGMATIC [38]	2202	Lung High CAT risk	Dalteparin 5000 IU/24 h 24 weeks vs no thromboprophylaxis	Overall survival HR, 1.01; *p* = 0.814	5.5% vs 9.7% *p* = 0.001	1.1% vs 0.7% *p* = NS	Clinically relevant non-major bleeding 4.5% vs 0.6%
RASTEN [39]	390	Small-cell lung cancer High CAT risk	Enoxaparin 1 mg/kg/24 h, 3 months, until a change of CT regimen vs no thromboprophylaxis	Overall survival HR, 1.11; *p* = 0.36	2.7% vs 8.4% *p* = 0.02	14% vs 3%	NR
AVERT [40]	574	Any location. Khorana ≥ 2	Apixaban 2.5 mg twice daily) for 6 months vs no thromboprophylaxis	Thrombosis-related, mITT analysis	4.2% vs 10.2% *p* < 0.001	mITT analysis: 3.5% vs 1.8% *p* = 0.046 Treatment-period analysis: 2.1% vs 1.1% *p* = NS	Clinically relevant non-major bleeding 7.3% vs 5.5%, *p* = NR
CASSINI [41]	841	Any location. Khorana ≥ 2	Rivaroxaban 10 mg daily for 6 months vs no hromboprophylaxis	Thrombosis-related, ITT analysis	ITT analysis (primary endpoint): 6.0% vs 8.8%, *p* = 0.10 Intervention-period analysis 2.6% vs 6.4%; HR 0.40, 95% CI 0.20–0.80	Intervention-period analysis 2.0% vs 1.0%, HR 1.96, 95% CI 0.59–6.49	Clinically relevant non-major bleeding 2.72% vs 1.98%, *p* = 0.53
TARGET-TP [42]	328	Lung and gastrointestinalcancers High CAT risk	Enoxaparin, 40 mg,daily for 90 days (extending up to 180 days according to ongoing risk) vs no thromboprophylaxis	Thrombosis-related	8% vs 23% *p* = 0.005	1% vs 2% *p* = 0.88	Clinically relevant non-major bleeding 16% vs 9%; HR 0.68 95% CI 0.30–1.55

n: sample size, *CAT* cancer-associated thrombosis, *MB* major bleeding, *vs* versus, *CT* chemotherapy, *p* significance level, *HR* hazard ratio, *CI* confidence interval, *ITT* intention to treat, *mITT* modified intention to treat, *VTE* venous thromboembolism, *ATE* arterial thromboembolism, *NS* no significant, *NR* not reported

Since the publication of the previous guidelines, several RAMs such as the ONCOTEV scale, the New-Vienna CATS scale, and the TiC Onco scale have been revalidated [43–45], with this last one even reaching a higher predictive value than the Khorana score. Moreover, a new scale published by Li et al. in 2023 has been developed and validated using artificial intelligence [46].These studies increase the value of these scales for assessing thrombotic risk in ambulatory oncological patients undergoing systemic treatment by identifying those who will benefit the most from thromboprophylaxis and, conversely, avoiding the risk of bleeding in those who do not require it.

A new RCTs in ambulatory thromboprophylaxis has been published since the last guide update. The TARGET-TP trial evaluated thromboprophylaxis in lung and colorectal cancer patients stratifying the risk of VTE based on fibrinogen and d-dimer levels: low-risk patients were observed, and high-risk patient were randomised to receive enoxaparin 40 mg subcutaneously daily for at least 90 days or no thromboprophylaxis (control arm). VTE occurred in 8% of high-risk patients randomised to enoxaparin vs 23% of patients in the control arm [hazard ratio (HR) 0.31; 95% CI 0.15–0.70; *p* = 0.005], and 8% of low-risk patients. No differences in MB rate were observed between the three groups: 1% in high-risk enoxaparin group, 2% in high-risk observation group, and 2% in low-risk patients (*p* = 0.88). Interestingly, mortality at 8 months was 13% in the enoxaparin group vs 26% in the high-risk control group (HR 0.48; 95%CI 0.24–0.92; *p* = 0.03), and 7% in the low-risk group [42].

Five new meta-analyses and two pre-planned subgroup analyses of RCTs relating to primary thromboprophylaxis in cancer outpatients with solid tumours receiving chemotherapy have been published since the SEOM’s last VTE guidelines. Three meta-analyses included the assessment of LMWH and DOACs compared to placebo or standard of care. Two meta-analyses also included warfarin, and one included only LMWH. All these meta-analyses except one found moderate-to-strong evidence of reduced incidence of VTE following thromboprophylaxis, both with LMWHs and with DOACs, but without a significant increase in the risk of MB. Some meta-analyses examined the impact on survival but, with the exception of Babarawi et al., did not find any statistically significant differences [47–51].

During this period, a meta-analysis and a pre-planned subgroup analysis of the CASSINI study focussing on the effects of thromboprophylaxis on pancreatic cancer patients were published. The pre-planned study showed that rivaroxaban did not result in a significantly lower incidence of VTE or VTE-related deaths in the 180-day period. However, during the intervention period, rivaroxaban substantially reduced VTE without increasing MB [52]. Frere et al. published a meta-analysis of five RCTs with 1003 pancreatic cancer patients [53]. Compared to placebo, thromboprophylaxis significantly decreased the risk of VTE (pooled RR 0.31; 95% CI 0.19–0.5; *p* < 0.00001), with an estimated number of 11.9 patients needed to treat to prevent one VTE event. Similar reductions were observed in studies with parenteral (RR 0.30; 95% CI 0.17–0.53) vs oral anticoagulants (RR 0.37; 95% CI 0.14–0.99). The pooled RR for MB was 1.08 (95% CI 0.47–2.52; *p* = 0.85).

Another meta-analysis focussing on lung cancer patients (with any histology and at any stage) receiving chemotherapy was conducted to determine the impact of primary ambulatory thromboprophylaxis with LMWHs on overall survival and the incidence of VTE. The analysis showed no survival benefit. However, there was a benefit in the incidence of VTE (RR 0.54; 95% CI 0.43–0.69; *p* < 0.00001) [54]. Moreover, the association of ALK/ROS1 rearrangements in non-small cell lung cancer (NSCLC) and a higher incidence of venous thrombosis has been described in several cohorts [55, 56]. However, no RCTs to verify whether venous thromboprophylaxis in this subgroup of patients contributes to a reduction in the risk of VTE compared to placebo or standard of care have been conducted.

Further considerations may involve the use of thromboprophylaxis with apixaban or rivaroxaban in specific cancer types, such as those of the gastrointestinal (GI) or genitourinary (GU) tract, demonstrating a higher susceptibility to bleeding in RCTs with DOACs [40, 47].

There is still a need for studies that provide information on the interactions between DOACs and cancer-specific drugs. DOACs are therefore not recommended for concomitant use with potent P-glycoprotein and CYP3A4 inhibitors or inducers [57].

Cancer patients are usually unaware of the risk of VTE. It is important that health care providers, including physicians and nurses, perform periodic risk assessment and educate patients about risk factors and early symptoms and sings of VTE. It is recommended to discuss the indication for pharmacological thromboprophylaxis and its potential risks and benefits with the patient [58, 59].

#### Recommendations

According to our systematic review, new studies have been published during the period 2020–2023, including meta-analyses and RCTs, which provide additional evidence supporting the recommendations of the previous guidelines.

The emergence of new data has led to subtle improvements to the recommendations:

- Assessment of VTE risk in cancer patients in the outpatient setting is recommended at initiation of systemic therapy and during the evolution of the disease. It is recommended to use a validated RAM to assess VTE risk (level of evidence: I; grade of recommendation: A).
- Routine primary thromboprophylaxis is not recommended in ambulatory patients with cancer (level of evidence: I; grade of recommendation: A).
- Primary pharmacological thromboprophylaxis of VTE with LMWH (level of evidence: I; grade of recommendation: A) or DOACs (level of evidence: I; grade of recommendation: B) is indicated in ambulatory patients who are receiving systemic anticancer therapy and are at an intermediate-to-high risk of VTE as assessed by a validated RAM and not at high risk of bleeding. Thromboprophylaxis should be continued for at least 12 weeks (level of evidence: I; grade of recommendation: B). If DOACs are chosen, a specific drug-drug interaction assessment should be done (level of evidence: IV; grade of recommendation: C).
- Primary pharmacological thromboprophylaxis of VTE with LMWH or DOACs may be appraised individually in ambulatory patients with locally advanced or metastatic pancreatic cancer (level of evidence: I; grade of recommendation: A) or advanced NSCLC harbouring ALK or ROS1 rearrangement treated with systemic anticancer therapy who are at low risk of bleeding (level of evidence: III; grade of recommendation: C).
- Health care providers should educate patients and make them aware of the risk factors, early symptoms and signs of VTE when initiating the therapy and during the evolution of the disease (level of evidence: III; grade of recommendation: A).

### Treatment

The main goals of anticoagulation therapy in CAT are to improve acute and subacute symptoms, reduce future sequelae, such as post-thrombotic syndrome or chronic thromboembolic pulmonary hypertension, and minimise the recurrence rate of VTE with the lowest possible risk of MB.

1. Treatment of CAT

The treatment of thrombosis can usually be divided into: acute phase (up to 10 days after diagnosis), long-term phase (first 3–6 months) and extended phase (beyond 6 months).

- 1a. Initial treatment of VTE in cancer patients (up to 10 days)

Based on a meta-analysis, LMWH may reduce recurrent VTE and mortality with similar risk of MB compared with UFH [60]. However, fondaparinux has not been shown to statistically improve the recurrence of VTE, bleeding and mortality compared to LMWH or UFH. As a result, its use is relegated to special situations [61].

Three studies have demonstrated that DOACs, apixaban and rivaroxaban are non-inferior to LMWH in terms of VTE recurrence and could provide an alternative option in the initial treatment of CAT [62, 63].

#### Recommendations

- LMWH or DOACs —rivaroxaban and apixaban— could be used for the acute phase of CAT. The choice of treatment must be individualised after careful consideration of bleeding risk and drug-drug interactions (level of evidence: I; grade of recommendation: A).
- UFH, and vitamin K antagonist (VKA) in cases of renal impairment (creatinine clearance < 30 mL/min) and fondaparinux in heparin-induced thrombocytopenia history, can be considered alternative agents (level of evidence: I; grade of recommendation: B).

- 1b. Long-term phase (3–6 months)

Historically, LMWH has been the treatment of choice for CAT. Several trials and meta-analyses have demonstrated the superiority of LMWH over VKA in terms of VTE recurrence, with no difference in MB events and mortality and with a reduction in CRNMB [64].

To date, five RCTs have supported the efficacy of DOACs in the treatment of CAT (SELECT-D, HOKUSAI-VTE, ADAM-VTE, Caravaggio, CASTA-DIVA) [62, 63, 65–67] (Table 4). The lack of head-to-head comparisons prevents any particular DOAC from being recommended over the others.

**Table 4:** Randomised clinical trials (RCTs) of direct-acting oral anticoagulants (DOACs) vs low-molecular weight heparins (LMWH) for the treatment of cancer-associated thrombosis (CAT)

Study	n	DOAC regimen and duration	Primary endpoint	Recurrent VTE DOAC vs LMWH	MB DOAC vs LMWH	CRNMB DOAC vs LMWH	Mortality DOAC vs LMWH
SELECT-D trial [62] Pilot study	406	Rivaroxaban 15 mg twice daily for 3 weeks, then 20 mg once daily vs dalteparin (CLOT regimen) for 6–12 months	Recurrent VTE at 6 months	4% vs 11%, HR 0.43, 95% CI 0.19–0.99; *p* = NR	6% vs 4%, HR 1.83, 95% CI 0.68–4.96; *p* = NR	13% vs 4%, HR 3.76, 95% CI 1.63–8.69; *p* = NR	25% vs 30%, *p* = NR
HOKUSAI-VTE [65] (non-inferiority)	1046	LMWH for 5 days, then edoxaban 60 mg daily vs dalteparin (CLOT regimen) for 6 months, up to 12 months Doses of 30 mg daily if: • CrCl = 30–50 mL/min·1.75 m^2^ • Body weight < 60 kg • Concomitant potent P-glycoprotein inhibitors	Composite of recurrent VTE or MB at 12 months 13.5% vs 12.8%; HR 0.97, 95% CI 0.7 to 1.36, *p* = 0.006 for non-inferiority; *p* = 0.87 for superiority	7.9% vs 11.3%, HR 0.71, 95% CI 0.48–1.06; *p* = 0.09	6.9% vs 4.0%, HR 1.77, 95% CI 1.03–3.04; *p* = 0.04	14.6% vs 11.1%, HR 1.38, 95% CI 0.98–1.94; *p* = NR	39.5% vs 36.6%, HR 1.12, 95% CI 0.92–1.37; *p* = NR
ADAM-VTE [66]	300	Apixaban 10 mg twice daily for 7 days, then 5 mg twice daily vs dalteparin (CLOT regimen) for 6 months	MB at 6 months	0.7% vs 6.3%, HR 0.09, 95% CI 0.013–0.780; *p* = 0.028	0% vs 1.4%, (HR not estimable)	6.2% vs 6.3%, HR 0.93, 95% CI 0.41–1.94; *p* = 0.88	16% vs 11%, HR 1.40, 95% CI 0.82–2.43; *p* = 0.31
CARAVAGGIO [63] (non-inferiority)	1170	Apixaban 10 mg twice daily for 7 days, then 5 mg twice daily vs dalteparin (CLOT regimen) for 6 months	Recurrent VTE at 6 months	5.6% vs 7.9%, HR 0.63, 95% CI 0.37–1.07; *p* < 0.001 for non-inferiority; *p* = 0.09 for superiority	3.8% vs 4.0%, HR 0.82, 95% CI 0.40–1.69; *p* = 0.60	9.0% vs 6.0%, HR 1.42, 95% CI 0.88–2.30; *p* = NR	23.4% vs 26.4%, *p* = NR
CASTA DIVA [67](non-inferiority)	158	Rivaroxaban 15 mg twice daily for 3 weeks, then 20 mg once daily vs dalteparin (CLOT regimen) for 3 months	Recurrent VTE at 3 months	6.4% vs 10.1%, SHR 0.75, 95% CI 0.21–2.66	1.4% vs 3.7%, SHR 0.36, 95% CI 0.04–3.43	12.2% vs 9.8%, SHR 1.27, 95% CI 0.49–3.26	25.7% vs 23.8%, HR 1.05, 95% CI 0.56–1.97

*CAT* cancer-associated thrombosis, *CI* confidence interval, *CrCl* creatinine clearance, *CRNMB* clinically relevant non-major bleeding, *HR* hazard ratio, *MB* major bleeding, *n* sample size, *NR* not reported, *p* significance level, *SHR* subdistribution hazard ratio, *VTE* venous thromboembolism

CLOT regimen: subcutaneous dalteparin at a dose of 200 IU/kg of body weight once daily for 1 month, followed by dalteparin at a dose of 150 IU/kg once daily for 2-6 months. Composite bleeding endpoint (MB plus CRNMB)

All the above-mentioned RCTs proposed dalteparin as a comparator arm (CLOT regimen) [68]. The treatment was administered for 6 months in all of them except the CASTA-DIVA trial [67], which was based on only 3 months. All the trials included symptomatic and incidental events. Two facts that are particularly worth noting are that the only study that included splanchnic visceral thrombosis (SVT) and upper extremity DVT was the ADAM-VTE trial, and that the Caravaggio trial excluded patients with primary brain tumour and brain metastases.

Regarding the objectives under evaluation, the SELECT-D, Caravaggio and CASTA-DIVA trials considered recurrent VTE as the primary endpoint. SELECT-D reported a significant reduction of VTE recurrence with rivaroxaban (HR 0.43; 95% CI 0.19–0.99), with more MB and CRNMB, particularly in upper GI tract tumours, being subsequently excluded from enrolment as a precautionary measure [62]. The Caravaggio study similarly proved that apixaban was non-inferior to dalteparin for the treatment of CAT (*p* < 0.001 for non-inferiority) without an increased risk of MB and CRNMB [63]. The HOKUSAI-VTE trial indicated that edoxaban was non-inferior to dalteparin in relation to a composite endpoint of recurrent VTE and MB at 12 months. A 3.4% difference in recurrent VTE favouring edoxaban was observed, whereas a significant 2.9% increase in MB events was also attributed to it [65]. Finally, the ADAM-VTE trial proposed MB events as the primary endpoint, rendering no differences in relation to this or CRNMB events (6% in both arms). As for recurrent VTE, a secondary endpoint in this trial, there was a significant reduction for apixaban (HR 0.099; 95% CI 0.013–0.780; *p* = 0.0281) [66]. The results of the CASTA-DIVA trial are not interpretable due to having an insufficient number of patients to reach the predefined criteria for non-inferiority, but the efficacy and safety results with rivaroxaban were consistent with those previously reported [67]. The CANVAS study, a non-inferiority RCT comparing LMWH and DOACs, has just been reported [69]. Physicians and patients selected any DOACs, including edoxaban, apixaban, rivaroxaban or dabigatran, or any LMWH. Considering recurrent VTE at 6 months as the primary endpoint, the use of any DOAC was found to be non-inferior to LWMH. Several limitations raise concerns about the results of the trial: the late randomisation (within 30 days from diagnosis) and the lower adherence rate for the LMWH arm may have affected the results. As no studies have evaluated the use of dabigatran in CAT to date, no formal recommendation about its use can be made.

Several meta-analyses comparing DOACs with LMWH have been conducted. Whilst most of them reported a significant decrease in recurrent VTE compared to dalteparin, conflicting results arise when assessing MB events, with no differences in mortality [70–75].

A common concern since the first evidence of the use of DOACs in CAT was found has been the risk of haemorrhage, especially in non-resected GU/GI tumours. The prevalence of GU tumours in the populations of DOAC trials varies between 2 and 15%, and is above 30% in the case of GI tumours [76]. In the SELECT-D trial, patients with gastroesophageal cancer tended to experience more major bleeds with rivaroxaban than with dalteparin (36% vs 11%) and, after an interim analysis, this type of tumour was excluded [62]. The same occurred in the HOKUSAI-VTE trial: patients with GI cancer had a higher rate of MB with edoxaban compared to dalteparin (12.7% vs 3.6%) [65]. However, in the Caravaggio trial there was no excess of major GI bleeding with apixaban, although the number of CRNMB events was higher in patients with GI tumours in the apixaban arm [77].

Cases of concomitant use of strong cytochrome P450 3A4 (CYP3A4) or P-glucoprotein inhibitors/inducers are another situation in which LMWH could be preferable. Despite this concern, some data suggests that antineoplastic treatment can be safely administered when using DOACs. Specifically, in Caravaggio study, the efficacy and safety of apixaban and dalteparina showed no differences in patients treated or not with anticancer agents. There were also no effects on recurrent VTE, MB or death with p-glyoprotein and/or CYP3A4 inhibitors or inducers [78].

As for quality of life, the ADAM-VTE trial reports that apixaban arm subjects had a better quality of life and adherence to treatment compared to the dalteparin arm [66]. Several clinical trials are currently testing the role of different monoclonal antibodies, such as factor XI inhibitors, in CAT [79].

Despite this evidence, DOACs are still awaiting financial approval from the health authorities in Spain.

#### Recommendations

- LMWH and DOACs —apixaban, edoxaban and rivaroxaban— are the preferred options for the long-term phase of CAT. The choice of treatment must be individualised after careful consideration of bleeding risk and drug-drug interactions (level of evidence: I; grade of recommendation: A).
- In patients with GI/GU tumours with a high bleeding risk, LMWH may be the first option. Apixaban seems to have a better safety profile compared to other DOACs and could be an alternative option (level of evidence: I; grade of recommendation: B).

- 1c. Extended phase (beyond 6 months)

The exact duration of treatment for CAT is unknown. Whereas most studies propose 3 to 6 months, others suggest that the risk of thrombotic complications is still present beyond 6 months [80, 81]. Published studies reporting events occurring 6 to 12 months after index VTE place the incidence of recurrent VTE at between 1 and 12%, with MB events between 2 and 5% [82]. Several factors to predict the risk of recurrent thrombosis have been proposed, such as the index DVT [83] or D-dimer or C-reactive protein levels 3 weeks after the discontinuation of anticoagulation [83, 84].

In the HOKUSAI-VTE study, approximately half of the patients continued therapy beyond 6 months. A post-hoc analysis showed that the rates of recurrent VTE or MB are relatively low and concluded that extended treatment with edoxaban appears as effective and safe as dalteparin [85].

The SELECT‐D 12 m trial tested extended anticoagulation in patients with active cancer and residual DVT/PE beyond 6 months and was underpowered to detect a statistically significant reduction in recurrent VTE [86].

The ONCO DVT trial randomised cancer patients with isolated DVT to receive 3 vs 12 months of edoxaban and observed a statistically significant reduction in the composite endpoint of recurrent VTE and VTE-related death (OR 0.13; 95% CI 0.03—0.44) at 12 months, with no differences in MB events (OR 1.34; 95% CI, 0.75—2.41), favouring the extended treatment [87].

#### Recommendations

- Therapeutic anticoagulation is recommended for 6 months (level of evidence: I; grade of recommendation: A).
- Extended anticoagulation beyond 6 months should be provided according to the benefit-risk balance in each patient (high risk of CAT recurrence, risk of bleeding complications, active cancer, systemic therapy and patients preference). In addition, the benefit-risk profile should be assessed periodically (level of evidence: II; grade of recommendation: C).

2. Incidental CAT

There are no changes to our previous statement regarding the management of incidental and symptomatic thrombosis.

In the Caravaggio trial cancer patients with incidental VTE (iVTE) showed a considerable risk of recurrent VTE, detecting it in a 4.3%, compared with 7.4% of those who initially presented an symptomatic VTE (sVTE) (HR 0.57; 95%CI 0.29–1.10). MB occurred in 5.2% of patients with iVTE and in 3.6% of patients with sVTE (HR 1.43; 95%CI 0.72–2.77) [88].

A recent post-hoc analysis of prospective studies in patients with CAT has reported no significant differences in the rate of VTE recurrence between incidental and symptomatic events (0.4 per 100 patients/month vs 0.5 per 100 patients/month; *p* = 0.313). However, the cumulative incidence of clinically relevant bleeding was significantly higher in patients with iVTE (7.9% vs 4.4%; OR 1.8; 95% CI 1.01–3.2) when compared with sVTE [89].

The available data in incidental SVT are scarce. Two international registries concluded that the prognosis of incidental SVT was similar to symptomatic SVT, and anticoagulant treatment appeared to be associated with a lower risk of recurrent VTE (HR 0.42; 95% CI 0.27–0.64) without increased MB risk [90, 91].

#### Recommendations

- Symptomatic and incidental cases of CAT should be treated equally, with LMWH or DOACs recommended as standard treatment (level of evidence: I; grade of recommendation: A).
- Treatment of isolated, incidental subsegmental PE or incidental SVT should be individualised in each patient according to the risks and benefits of anticoagulant treatment. Clinicians might suggest considering anticoagulation in these cases (level of evidence: II; grade of recommendation: C).

### Prevention and treatment of vte in special cancer situations

1. Prophylaxis and treatment of central venous catheter-related thrombosis (CVCrT)

The incidence of CVCrT varies between studies, depending on whether it is asymptomatic (2–66%) or symptomatic (2.7–13.8%) and the diagnostic method used. The material used, the use of peripherally inserted central catheters (PICCs) over implantable ports (IP) (the incidence of VTE was around five times higher with a PICC vs IP [11% vs 2%]), and insertion and placement technique can all raise the risk of CVCrT [92, 93].

The CVC should be placed on the right side, specifically in the jugular vein, with the distal tip placed at the junction of the superior vena cava and the right atrium [94].

Several studies, including one meta-analysis, do not support the use of routine thromboprophylaxis for CVC in cancer patients [92]. A Cochrane [64] review concluded that, compared with no prophylaxis, LMWH reduces CVCrT with no increase in bleeding risk, whereas VKA may reduce the incidence of CVCrT but whilst increasing the risk of bleeding.

Regarding treatment of CVCrT, the evidence remains scant and comes from extrapolation from CAT clinical trials and from some retrospective studies and one prospective study [95, 96]. LMWH is the main treatment, and a minimum treatment of 3–6 months is needed. Indefinite treatment should be considered if the catheter is not removed, and the cancer is present. The CVC should only be removed if its use is no longer needed or it is infected, if there is a contraindication to anticoagulant treatment or if the thrombus progresses despite anticoagulation, and it should be done after 5–7 days of treatment [97]. DOACs can be considered in the treatment of CVCrT. The Catheter2 [98] and Catheter3 [99] studies showed high preservation of CVC function after treating CVCrT with rivaroxaban or apixaban, respectively. However, these drugs have not been approved for the treatment of CAT by the Spanish health authorities.

#### Recommendations

- Routine pharmacological prophylaxis of CVCrT is not recommended (level of evidence: I; grade of recommendation: A).
- Catheters should be placed on the right side, in the jugular vein, and the distal tip should be located at the junction of the superior vena cava and the right atrium (level of evidence: I; grade of recommendation: B).
- For the treatment of symptomatic CVCrT, anticoagulant treatment is recommended for a minimum of 3 months (level of evidence: III; grade of recommendation: A). LMWH is suggested, although VKA or DOACs may be considered as alternative options (level of evidence: III; grade of recommendation: C).
- It is recommended to remove the catheter if it is not needed or it is infected, if anticoagulant treatment is contraindicated or if there is clinical deterioration due to thrombus extension despite treatment (level of evidence: III; grade of recommendation: B).
- In patients with CVCrT who have completed 3 months of anticoagulant treatment, extended anticoagulation until catheter removal is suggested if the patient’s bleeding risk is low (level of evidence: IV; grade of recommendation: B).

2. VTE treatment of central nervous system (CNS) primary tumours and metastases

The presence of a stable or active primary intracranial malignancy or brain metastasis is not an absolute contraindication to anticoagulation, but the risk of intracranial haemorrhage (ICH) is always a concern. No prospective studies looking at the incidence of ICH with therapeutic anticoagulation with protocol-scheduled imaging have been reported. A recent RIETE registry publication shows that patients with active brain cancer have a higher risk of ICH and fatal ICH during anticoagulant treatment than patients with non-brain cancer or those without cancer [100].

Most brain tumours, adequately treated, do not pose an excessive risk of bleeding in VTE patients who are otherwise appropriate candidates for anticoagulation treatment. Possible exceptions are brain metastases from melanoma, choriocarcinoma, thyroid carcinoma and renal cell carcinoma, which have a higher rate of spontaneous intra-tumour bleeding than other tumour types.

Limited data from a meta-analysis [101] that included nine retrospective cohort studies of patients with brain metastases or primary glioma treated with LMWH and/or warfarin suggests that therapeutic anticoagulation does not increase ICH risk amongst patients with brain metastases but may increase risk amongst patients with primary brain tumours. Giustozzi et al. [102], in a meta-analysis that included thirty studies, found that patients with brain metastases had a particularly high risk of ICH compared to primary brain cancers (13% vs 6.4%; RR 3.26; 95%CI 2.69–3.94). During anticoagulant treatment, patients with primary brain tumours had a higher risk of ICH, compared with nonanticoagulated patients (12.5% vs 4.4%; RR, 2.63; 95% CI, 1.48–4.67; I25 49.6%). DOACs were associated with a lower risk of ICH than LMWH.

On the other hand, a recent meta-analysis covering six articles and including 566 patients with brain tumours [103] demonstrated that DOACs are associated with a lower risk of ICH than LMWH therapy in the treatment of VTE associated with brain tumours, especially in patients with primary brain tumours (RR 0.18; 95% CI 0.06–0.50; *p* = 0.001). However, there were no statistical differences between the two treatments for metastatic brain tumours or in terms of lethal ICH.

#### Recommendations

- For patients with brain metastases or primary brain tumours, in the absence of contraindications, complete anticoagulation is recommended for VTE treatment (level of evidence: II; grade of recommendation: B).
- Treatment with DOACs may be a reasonable option for treating VTE in patients with primary brain tumours given the tentative evidence of lower rates of ICH (level of evidence: IV; grade of recommendation: C).

3. VTE treatment and prophylaxis in patients with cancer and with coronavirus disease 2019 (COVID-19)

During the COVID-19 pandemic, it was observed that SARS-CoV-2 (severe acute respiratory syndrome coronavirus 2) infection promotes a prothrombotic state in infected patients, leading to a higher incidence of VTE, especially amongst hospitalised patients [104–106]. However, there are few studies discussing the incidence of these thromboembolic events in patients with active cancer who are infected with SARS-CoV-2 [107].

The limited evidence does not currently suggest a correlation between the presence of SARS-CoV-2 infection, or its severity, as a risk factor for a higher incidence of thrombosis in patients with both cancer and COVID-19 [108–110].

The randomised studies conducted with tinzaparin or bemiparin on the general population, with limited representation of cancer patients, have not demonstrated any benefit from increasing the dosage of LMWH from a prophylactic to an intermediate or therapeutic dose [111, 112]. On the other hand, a decreased risk of VTE was demonstrated in another study conducted with thromboprophylactic rivaroxaban during the 35 days following hospital discharge [113].

#### Recommendations

- In the management of active cancer patients with COVID-19, we recommend following the same directions as for any hospitalised or outpatient individual diagnosed with active cancer (level of evidence: II; grade of recommendation: C).

4. Renal impairment

As chronic kidney disease is common in patients with cancer (up to 50–60%) [114], all patients should have a measure of estimated glomerular filtration rate (eGFR) calculated using the Cockcroft-Gault formula [115]. Patients with VTE and concomitant renal impairment are at higher risk of MB and recurrent VTE during anticoagulant treatment compared to patients with normal renal function.

Patients with mild or no renal impairment (eGFR > 50 mL/min) should be fully anticoagulated.

The CLOT [68] and CATCH [116] trials in CAT, using dalteparin or tinzaparin, had a small number of patients with moderate renal impairment (creatinine clearance [CrCl] = 30–60 mL/min; CLOT: 24%; and CATCH: 15%) in whom MB was comparable to that of patients with normal renal function on LMWH [116]. Whilst LMWH may produce less bleeding, the use of DOACs is also possible, since subgroup analyses suggest that, in patients with CAT and moderate renal impairment (CrCl = 30–60 mL/min), the efficacy and safety of LMWH and DOACs are generally consistent with those of cancer patients without renal impairment [117].

Patients with CAT and severe renal impairment (CrCl < 30 mL/min) were excluded from the pivotal trials on the treatment of CAT. These patients can be treated with UFH followed by VKA. The other option is using LMWH with the dose adjusted to the anti-Xa activity level (Table 5) if available. In RCTs of DOACs, patients with severe renal impairment were excluded.

**Table 5:** Adjusted doses of low-molecular-weight heparin (LMWH) in cases of severe renal insufficiency

Drug	eGFR < 30 mL/min
Bemiparin	Adjust the dose to 75%. Anti-Xa activity monitoring
Enoxaparin	1 mg/kg/24 h. Anti-Xa activity monitoring
Tinzaparin	Does not accumulate with CrCl > 20 mL/min. Anti-Xa activity monitoring

*CrCl* creatinine clearance, *eGFR* estimated glomerular filtration rate

#### Recommendations

- For patients without or with mild to moderate renal impairment (eGFR ≥ 30 mL/min), full doses of anticoagulant—LMWH or DOACs—can be used (level of evidence: I; grade of recommendation: B).
- For patients with severe renal impairment (eGFR < 30 mL/min), consider using UFH/VKAs or renal dosing of LMWH with anti-Xa monitoring (level of evidence: V; grade of recommendation: C).

5. Extreme weights

The treatment of VTE in obese cancer patients has not been extensively studied. Some evidence is provided by three studies conducted in the general population using dalteparin, enoxaparin and tinzaparin. These studies suggest that, in obese patients (> 120 kg or body mass index [BMI] > 40 kg/m^2^), the dose to be administered in the treatment of VTE should correspond to the patient's actual weight, without capping the dose [118–120]. Regarding the use of new DOACs, there are no robust studies available. However, there is also no reported data indicating that this therapy is ineffective or unsafe in this population [121].

In thromboprophylaxis for patients with a BMI > 40 kg/m^2^, studies carried out on the general population have found that increasing the dose of dalteparin to 7500 IU or 5000 IU every 12 h is effective and safe [122, 123], and the same result has been found when increasing the dose of enoxaparin to 40 mg every 12 h or 0.5 mg/day (even considering an increase to 60 mg every 12 h for BMI > 50 kg/m^2^) [124, 125].

As for thromboprophylaxis in patients with extreme low body weight (25–50 kg), there is limited data available. However, maintaining the same standard doses has been shown to potentially lead to overdosing and reduced safety in the general population. Therefore, some studies have found lower doses of dalteparin (2500 IU/24 h or 100 IU/kg·24 h) or enoxaparin (20 mg/24 h for patients weighing 25–40 kg, or 30 mg/24 h for those weighing 41–50 kg, with no renal impairment) to be equally effective and safer in this population [126, 127]. There are no studies with other drugs.

#### Recommendations

- In obese patients with cancer (BMI > 40 kg/m^2^ or weight > 120 kg), anticoagulation for VTE treatment with LMWH or DOACs should be used with caution, with strict clinical monitoring and individualised treatment (level of evidence: V; grade of recommendation: C).
- Regarding thromboprophylaxis, in obese patients we recommend considering dose escalation based on the LMWH to be used (preferably enoxaparin 40–60 mg/12 h or 0.5 mg/kg·24 h, or dalteparin 7500 UI/24 h or 5000 UI/12 h), whilst individualising the bleeding risk for each patient (level of evidence: III; grade of recommendation: C). In cancer patients with a body weight of 25–50 kg, we recommend reducing the dose (dalteparin, enoxaparin) to prevent overdosage (level of evidence: III; grade of recommendation: C).

6. Patients with thrombocytopenia

Decreased platelet count is a common occurrence in oncological patients, caused by a mechanism of toxicity (chemotherapy-induced thrombocytopenia) or by a tumour infiltration of the bone marrow. The risk of bleeding increases under thrombocytopenia conditions, in spite of which the risk of thrombosis is not reduced [128–130].

This situation poses a clinical challenge in the management of oncological patients with VTE and thrombocytopenia, and an assessment of the risk of bleeding and recurrence of VTE must be carried out. The risk of bleeding must take into consideration factors such as the need for invasive procedures, aetiology of thrombocytopenia, and degree and duration of thrombocytopenia, as well as general comorbidities of the patient, such as renal or hepatic insufficiency, tumour location with vascular involvement, or a history of bleeding or coagulopathies.

Thrombocytopenia is defined as a platelet count below 100 × 10^9^/L. In general, anticoagulation is safe when the platelet count is > 50 × 10^9^/L (grade 1–2 by National Institutes of Health [NCI] Common Terminology Criteria for Adverse Events [CTCAE]), so anticoagulation should be maintained at full doses in such cases [130, 131]. However, in a post-hoc analysis of HOKUSAI-VTE Study Patell et al [132] found a higher risk of MB inpatients who had platelet counts < 100 × 10^9^/L and > 50 × 10^9^/L vs those white platelet counts > 100 × 10^9^/L (9.0% vs 4.0%, *p* = 0.02). Thrombocytopenia did not impact on recurrent VTE (9.8% vs 7.4%, *p* = 0.37), or overall mortality (21.8% vs 26.0%, *p* = 0.48). The authors concluded that mild thrombocytopenia (platelet count < 100 × 10^9^/L and > 50 × 10^9^/L) was associated with twice the risk of MB in patients receiving anticoagulation for CAT.

In a patient anticoagulated for a VTE and with thrombocytopenia < 50 × 10^9^/L (NCI CTCAE grade 3–4), the patient’s individualised risk of bleeding and rethrombosis, as well as the type of anticoagulant treatment currently being administered, should be assessed.

- At a high risk of recurrence of thrombosis (first month of VTE, high thrombus burden PE and proximal DVT), the patient should continue with full-dose anticoagulation, maintaining platelet counts of > 50 × 10^9^/L with blood product transfusions. The studies carried out with a combination of full-dose LMWH and platelet transfusion put the range at 40–50 × 10^9^/L. The results should therefore be taken with caution [133].
- At low risk of thrombosis recurrence (catheter-related thrombosis, distal DVT > 1 month since the event): adjustment of LMWH anticoagulation dose and use of LMWH over DOACs and VKA. The rationale for the use of parenteral anticoagulation over oral anticoagulation includes greater evidence of LMWH in this setting [128], higher bleeding rates with DOACs compared to dalteparin in CAT [62, 65], a shorter half-life and more feasible dose reduction [134]. Therefore, the prophylactic LMWH dose or a 50% dose reduction should be used in the presence of grade 3 thrombocytopenia (< 50 to 25 × 10^9^/L) and low-risk VTE [135].

In patients with grade 4 thrombocytopenia (< 25 × 10^9^/L), suspension of anticoagulation should be considered until the platelet count recovers to grade 3 or higher, with an individualised approach for each patient, taking into account the risk–benefit balance of recurrence of thrombosis and bleeding.

#### Recommendations

- Thrombocytopenia grade 1–2 (platelet count < 100 and ≥ 50 × 10^9^/L): we recommend therapeutic-dose parenteral or oral anticoagulation (level of evidence: I; grade of recommendation: B).
- Thrombocytopenia grade 3 (platelet count < 50 and ≥ 25 × 10^9^/L): we suggest the use of LMWH over DOACs or VKA (level of evidence: V; grade of recommendation: C).
- Thrombocytopenia grade 3 (platelet count < 50 and ≥ 25 × 10^9^/L) and high-thrombotic-risk CAT or acute CAT: we suggest continuing full-dose anticoagulation and increasing platelet counts by means of transfusion support (level of evidence: V; grade of recommendation: C).
- Thrombocytopenia grade 3 (platelet count < 50 and ≥ 25 × 10^9^/L) and low-thrombotic-risk CAT or chronic CAT: we suggest reducing the dose of LMWH to prophylactic level or a 50% dose (level of evidence: V; grade of recommendation: B).
- Thrombocytopenia grade 4 (platelet count < 25 × 10^9^/L): we suggest holding anticoagulation until recovery of platelet count over 25 × 10^9^/L, taking into consideration the risks of bleeding and rethrombosis in a case-by-case assessment (level of evidence: V; grade of recommendation: C).
- We recommend resuming the appropriate dose of anticoagulation as soon as allowed by the platelet count (level of evidence: V; grade of recommendation: B).

7. Treatment of recurrent VTE during anticoagulation therapy

The recurrence rate of VTE in anticoagulated oncology patients varies between 4 and 11% according to the latest RCTs including LMWH and DOACs as therapy, [62, 63, 65, 66]. The most important risk factors are tumour stage, the presence of metastasis or locally advanced primary tumour factors that could lead to a higher risk [78], histological type of tumour, primary site, oncological disease status (increased risk of progression), molecular characteristics and previous history of VTE. To date, there are no validated predictive models for an adequate risk assessment of patients, and there are no RCTs available to guide the management of these situations.

Regarding the risk of bleeding, at least three risk assessment models specific to patients anticoagulated for CAT have recently been published. The CAT-BLEED score was derived from a cohort that specifically included patients with cancer from the HOKUSAI-VTE study [136]. The B-CAT score was developed from a retrospective observational study, in a cohort of 15,749 patients who experienced VTE in the setting of active cancer with a 6-month follow-up [137]. The PredictAI study, based on Natural Language Processing and Machine Learning, developed two models, one for predicting recurrences and the other for predicting MB events, in cancer patients treated with anticoagulants in the first 6 months after VTE diagnosis. The bleeding score has been externally validated in an independent cohort of 2179 patients from the TESEO study, with statistical significance in the logistic regression (ROC-AUC = 0.59; 95% CI 0.53–0.65; *p* = 0.002) and randomised forest (ROC-AUC = 0.56; 95% CI = 0.51–0.62; *p* = 0.023) models [138, 139].

We therefore propose empirical management with a clinical approach for patients with recurrence whilst anticoagulated with a personalised approach.

In the case of anticoagulated oncological patients diagnosed with a recurrent thromboembolic event, we suggest checking for good therapeutic compliance with adequate adherence, as well as considering the possibility of drug or food interactions that may reduce the efficacy of the pharmacological agents used. The possibility of heparin-induced thrombocytopenia, usually in the first 10–14 days from the start of treatment with LMWH, must also be ruled out. The strategy for adjusting anticoagulation therapy in these patients should also consider the following characteristics: type of anticoagulant drug, time of tumour progression, possible additional risk factors that could trigger the event that can be corrected, risk of bleeding, reason for reducing the dose compared to a full dose, and an assessment of whether the thrombosis is in a new location or is a continuation of the previous one.

For patients diagnosed with VTE with subtherapeutic doses of LMWH, the recommendations are to increase the dose of LMWH or switch to DOACs. In the case of patients with adequate doses of LMWH, two retrospective studies support a 25% increase in the LMWH dose [140, 141]. Another possibility is to change to a 12-h dosing schedule or switch to DOACs, on the basis of a trend towards a decreased risk in VTE in the acute therapy setting reported in one study [142]. In the case of patients with DOACs, in the absence of studies providing further evidence, switching to LMWH is recommended based on expert opinion.

Patients who are anticoagulated with VKA should have their anticoagulation switched to LMWH or DOACs [143]. However, the number of patients in this situation should be very low given the minimal use of this drug in oncological patients.

#### Recommendations

- Patients anticoagulated with VKA or subtherapeutic doses of LMWH: we suggest the use of therapeutic doses of LMWH or switching to DOACs (level of evidence: II; grade of recommendation: B).
- Patients with therapeutic doses of LMWH: we suggest increasing the dose by 25% or changing to a 12-h dosing schedule or switching to DOACs (level of evidence: II; grade of recommendation: B).
- Patients with full-dose DOACs: switching to LMWH should be considered (level of evidence: III; grade of recommendation: C).

8. Anticoagulation to improve cancer survival

Preclinical studies [144] have shown a correlation between the haemostatic system and cancer development. Its activation promotes tumour progression and dissemination. Anticoagulation treatment could play an anti-tumour role.

Several studies have failed to confirm any significant survival benefit from adding LMWH, VKA or DOACs [145]. Recently, the TARGET-TP trial found that administration of prophylactic enoxaparin in lung and gastrointestinal cancer patients with-high risk of VTE by a biomarker-based approach, significantly reduced the incidence of thromboembolism and all-cause mortality within the 180-day trial period. Six-month mortality was 13% in the enoxaparin group vs 26% in the high-risk control group (HR, 0.48; 95% CI, 0.24–0.93; *p* = 03) and 7% in the low-risk group (vs high-risk control: HR, 4.71; 95% CI, 2.13–10.42; *p* < 0.001) [42].

New studies will be necessary to determine the value of anticoagulation in the survival of patients with CAT.

#### Recommendation

- Anticloagulant therapy should not be prescribed to enhance survival (level of evidence: I; grade of recommendation: B).

**Table:** 

Table of summary of recommendations
Prophylaxis of Vte
1. Prophylaxis of VTE in patients with cancer hospitalised for an acute medical illness
• In the absence of bleeding or other contraindications, pharmacological thromboprophylaxis should be considered in hospitalised cancer patients with acute medical illness or reduced mobility [I; B]
• The preferred agents are LMWHs, due to their favourable safety profile [I; B]
• At this time, the authors do not recommend extended thromboprophylaxis among hospitalised medically ill patients with cancer, highlighting the need for an individualised approach to management [II; E]
2. Prophylaxis of VTE in surgical cancer patients
• In the absence of active bleeding, high bleeding risk or other contraindications, all cancer patients undergoing major surgical intervention should be offered pharmacological thromboprophylaxis with LMWH, the preferred agents, or UFH [I; A]
• Prophylaxis should be started before surgery or as soon as possible in the post-operative period. Patients should receive at least 7–10 days of prophylaxis [I; A]
• Mechanical methods may be added to pharmacological prophylaxis in high-risk patients but should not be used as monotherapy unless pharmacological prophylaxis is contraindicated [II; C]
• Patients undergoing major open or laparoscopic abdominal or pelvic cancer surgery with high-risk features, such as restricted mobility, obesity or history of VTE, or with additional risk factors, should be considered for extended thromboprophylaxis with LMWH for up to 4 weeks [I; A]
• Alternatively, those patients who are candidates for extended thromboprophylaxis after surgery may be offered prophylactic doses of rivaroxaban or apixaban after an initial period of LMWH. Additional data from more RCTs are needed to strengthen this recommendation [II; B]
3. Prophylaxis of VTE in ambulatory cancer patients during systemic therapy
• Assessment of VTE risk in cancer patients in the outpatient setting is recommended at initiation of systemic therapy and during the evolution of the disease. It is recommended to use a validated RAM to assess VTE risk [I; A]
• Routine primary thromboprophylaxis is not recommended in ambulatory patients with cancer [I; A]
• Primary pharmacological thromboprophylaxis of VTE with LMWH [I; A] or DOACs [I; B] is indicated in ambulatory patients who are receiving systemic anticancer therapy and are at an intermediate-to-high risk of VTE as assessed by a validated RAM and not at high risk of bleeding. Duration of the thromboprophylaxis should be at least 12 weeks [I; B]. If DOACs are chosen, a specific drug-drug interaction assessment should be done [IV; C]
• Primary pharmacological thromboprophylaxis of VTE with LMWH or DOACs may be appraised individually in ambulatory patients with locally advanced or metastatic pancreatic cancer [I; A] or advanced NSCLC harbouring ALK or ROS1 rearrangement treated with systemic anticancer therapy who are at low risk of bleeding [III; C]
• Health care providers should educate patients and make them aware of the risk factors, early symptoms and signs of VTE at the time of initiating the therapy and during the evolution of the disease [III; A]
Treatment
1. Acute phase (up to 10 days)
• LMWH or DOACs —rivaroxaban and apixaban— could be used for the acute phase of CAT. The choice of treatment must be individualised after careful consideration of bleeding risk and drug-drug interactions [I; A]
• UFH, and vitamin K antagonist (VKA) in cases of renal impairment (creatinine clearance < 30 mL/min) and fondaparinux in heparin-induced thrombocytopenia history, can be considered alternative agents [I; B]
2. Long-term phase (3–6 months)
• LMWH and DOACs —apixaban, edoxaban and rivaroxaban— are the preferred options for the long-term phase of CAT. The choice of treatment must be individualised after careful consideration of bleeding risk and drug-drug interactions [I; A]
• In patients with high bleeding risk GI/GU tumours, LMWH may be the first option. Apixaban seems to have a better safety profile compared to other DOACs and could be an alternative option [I; B]
3. Extended phase (beyond 6 months)
• Therapeutic anticoagulation is recommended for 6 months [I; A]
• Extended anticoagulation beyond 6 months should be provided according to the benefit-risk balance in each patient (high risk of CAT recurrence, risk of bleeding complications, active cancer, systemic therapy and patients preference). In addition, the benefit-risk profile should be assessed periodically [II; C]
4. Incidental CAT
• Symptomatic and incidental cases of CAT should be treated equally, with LMWH or DOACs recommended as standard treatment [I; A]
• Treatment of isolated, incidental subsegmental PE or incidental SVT should be individualised in each patient according to risk–benefit of anticoagulant treatment. Clinicians might suggest considering anticoagulation in these cases [II; C]
Prevention and treatment of Vte in special cancer situations
1. Prophylaxis and treatment of CVC-related thrombosis (CVCrT)
• Routine pharmacological prophylaxis of CVCrT is not recommended [I; A]
• Catheters should be placed on the right side, in the jugular vein, and the distal tip should be located at the junction of the superior vena cava and the right atrium [I; B]
• For the treatment of symptomatic CVCrT, anticoagulant treatment is recommended for a minimum of 3 months [III; A]. LMWH is suggested, although VKA or DOAC may be considered as alternative options [III; C]
• It is recommended to remove the catheter if it is not needed or it is infected, if anticoagulant treatment is contraindicated or if there is clinical deterioration due to thrombus extension despite treatment [III; B]
In patients with CVCrT who have completed 3 months of anticoagulant treatment, extended anticoagulation until catheter removal is suggested if the patient’s bleeding risk is low [IV; B]
2. VTE treatment of CNS primary tumours and metastasis
• For patients with brain metastases or primary brain tumours, in the absence of contraindications, complete anticoagulation is recommended [II; B]
• Treatment with DOACs may be a reasonable option for treating VTE in patients with primary brain tumours given the tentative evidence of lower rates of ICH [IV; C]
3. VTE treatment and prophylaxis in patients with cancer and COVID-19
• In the management of active cancer patients with COVID-19, we recommend following the same directions as for any hospitalised or outpatient individual diagnosed with active cancer [II; C]
4. Renal impairment
• For patients without or with mild to moderate renal impairment (eGFR ≥ 30 mL/min), full doses of anticoagulant can be used. [I;B]
• For patients with severe renal impairment (eGFR < 30 mL/min), consider use of UFH/VKA or renal dosing of LMWH with anti-Xa monitoring [V; C]
5. Extreme weights
• In obese patients with cancer (BMI > 40 kg/m2 or weight > 120 kg), anticoagulation for VTE treatment with LMWH or DOACs should be used with caution, with strict clinical monitoring and individualised treatment [V;C)
• Regarding thromboprophylaxis, in obese patients we recommend considering dose escalation based on the LMWH to be used (preferably enoxaparin 40–60 mg/12 h or 0.5 mg/kg·24 h, or dalteparin 7500 UI/24 h or 5000 UI/12 h), whilst individualising the bleeding risk for each patient [III; C]. In cancer patients with a body weight of 25–50 kg, we recommend reducing the dose (dalteparin, enoxaparin) to prevent overdosage [III; C]
6. Patients with thrombocytopenia
• Thrombocytopenia grade 1–2 (platelet count < 100 and ≥ 50 × 10^9^/L): we recommend therapeutic-dose parenteral or oral anticoagulation [I; B]
• Thrombocytopenia grade 3 (platelet count < 50 and ≥ 25 × 10^9^/L): we suggest the use of LMWH over DOACs or VKA [V; C]
• Thrombocytopenia grade 3 (platelet count < 50 and ≥ 25 × 10^9^/L) and high-thrombotic-risk CAT or acute CAT: we suggest continuing full-dose anticoagulation and increasing platelet counts by means of transfusion support [V; C]
• Thrombocytopenia grade 3 (platelet count < 50 and ≥ 25 × 10^9^/L) and low-thrombotic-risk CAT or chronic CAT: we suggest reducing the dose of LMWH to prophylactic level or a 50% dose [V; B]
• Thrombocytopenia grade 4 (platelet count < 25 × 10^9^/L): we suggest holding anticoagulation until recovery of platelet count over 25 × 10^9^/L, taking into consideration the risks of bleeding and rethrombosis in a case-by-case assessment [V; C]
• We recommend resuming the appropriate dose of anticoagulation as soon as allowed by the platelet count [V; B]
7. Treatment of recurrent VTE during anticoagulation therapy
• Anticoagulated patients with VKA or subtherapeutic doses of LMWH: we suggest the use of therapeutic doses of LMWH or switching to DOACs [II; B]
• Patients with therapeutic doses of LMWH: we suggest increasing the dose by 25% or changing to a 12-h dosing schedule or switching to DOACs [II; B]
• Patients with full-dose DOACs: switching to LMWH should be considered [III; C]
8. Anticoagulation to improve cancer survival
• Anticoagulant therapy should not be prescribed to enhance survival [I; B]

*BMI* body mass index, *CAT* cancer-associated thrombosis, *CNS* central nervous system, *COVID-19* coronavirus disease 2019, *CrCl* creatinine clearance, *CVC* central venous catheter, *CVCrT* central venous catheter-related thrombosis, *DOAC* direct-acting oral anticoagulants, *eGFR* estimated glomerular filtration rate, *GI* gastrointestinal, *GU* genitourinary, *ICH* intracranial haemorrhage, *LMWH* low-molecular-weight heparin, *NSCLC* non-small cell lung cancer, *PE* pulmonary embolism, *RAM* risk assessment model, *RCT* randomised clinical trial, *SVT* splanchnic visceral thrombosis, *UFH* unfractionated heparins, *VKA* vitamin K antagonist, *VTE* venous thromboembolism.

## Data Availability

Not applicable.
